# Supplementary material for: Amphipathic Small Molecule AZT Compound Displays Potent Inhibitory Effects in Cancer Cell Proliferation
Source: Pharmaceutics. 2021 Dec 3;13(12):2071. doi: 10.3390/pharmaceutics13122071 (PMC8704889; doi:10.3390/pharmaceutics13122071)
Supplement: Supplementary file 1 [file pharmaceutics-13-02071-s001.zip › pharmaceutics-1458833-supplementary.pdf]

pharmaceutics

# Supplementary Materials: Amphipathic Small Molecule AZT Compound Displays Potent Inhibitory Effects in Cancer Cell Proliferation

**Pethaiah Gunasekaran, Ho Jin Han, Jung hoon Choi, Eun Kyoung Ryu, Nam Yeong Park, Geul Bang, Yeo Kyung La, Sunghyun Park, Kyubin Hwang, Hak Nam Kim, Mi-Hyun Kim, Young Ho Jeon, Nak-Kyun Soung and Jeong Kyu Bang**

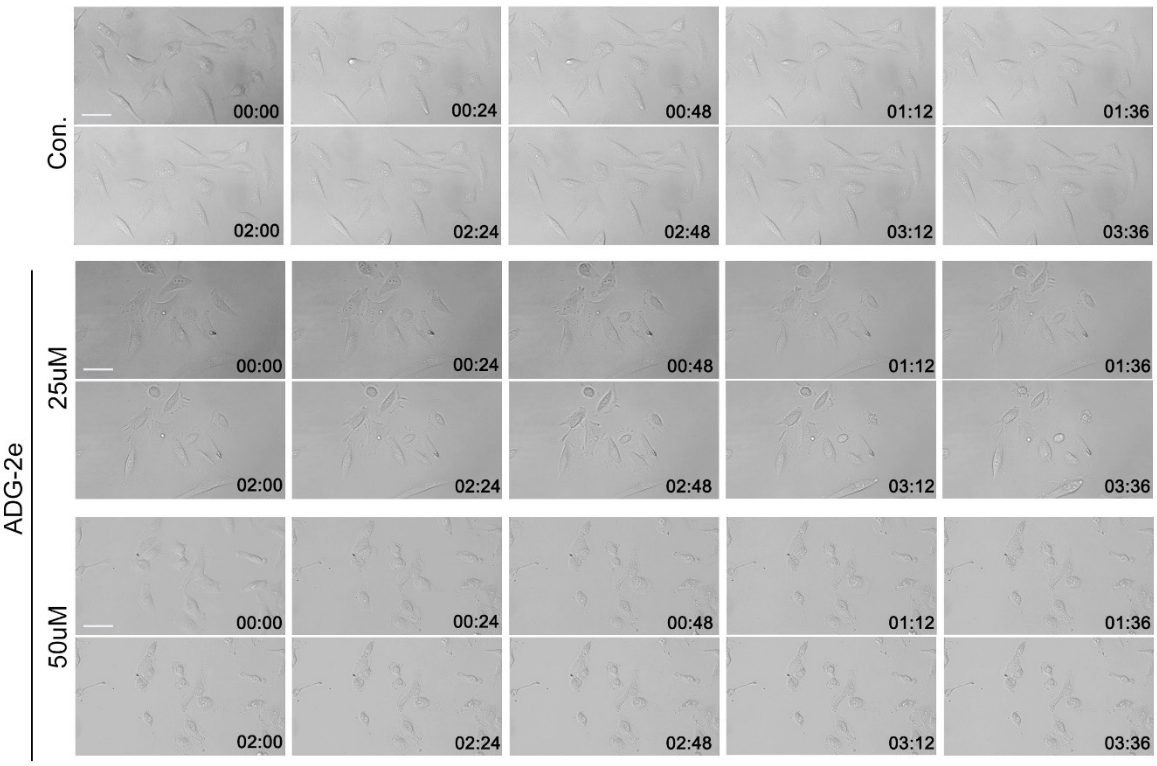


**Figure S1.** ADG-2e affects the cell membrane and ultimately destroys the cell membrane, leading to the cancer cells death. HeLa CCL2 cells were treated with ADG-2e at 25 or 50 μM, followed by observation. Each cell was observed for 3 hours at 12-minute intervals, and changes according to time were arranged according to time frame. Numbers are displayed in hours: minutes. Each still cut was selected from Movies #1, #2, and #3. The scale bar is 100 μm.

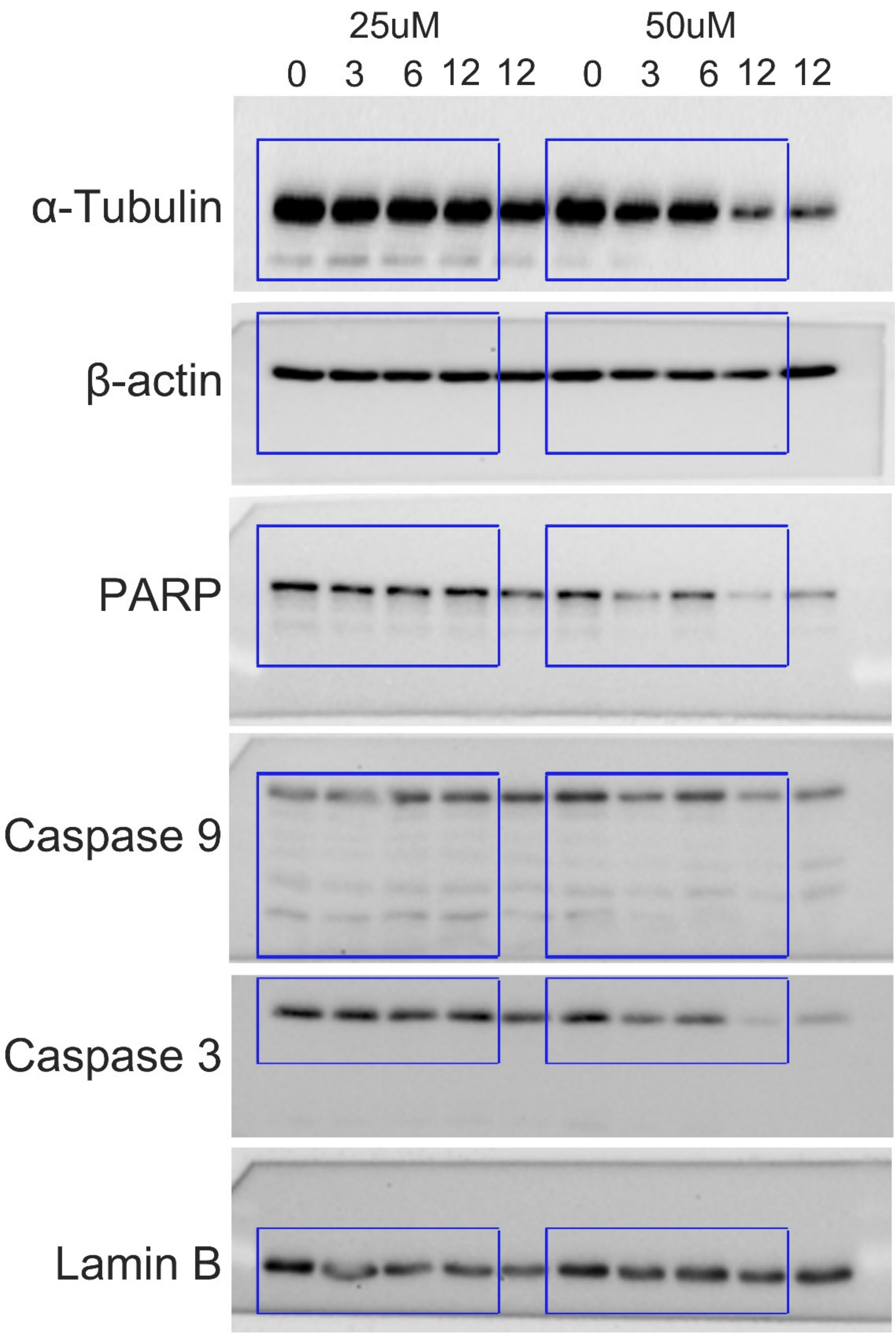


**Full length western blot analysis of fig 2c**

*Experimental procedure*

*Synthesis of ADG-2e [1]*

**1-((2R,4S,5R)-4-azido-5-(azidomethyl)tetrahydrofuran-2-yl)-5-methylpyrimidine-2,4(1H,3H)-dione (2)** To the stirred solution of 1 (1.0 g, 3.74 mmol) in dichloromethane (100 mL) at 0 °C, was added TEA (1.04 mL, 7.48 mmol), DMAP (0.045 g, 0.36 mmol) and tosyl chloride (0.7 g, 3.74 mmol) and stirred for 15 h at room temperature. Then the reaction mixture was diluted with dichloromethane (50 mL) and organic layer was washed with $NaHCO_3$ (20 mL) solution, brine $H_2O$ (20 mL) and dried over $Na_2SO_4$. Concentrated and flash chromatographic separation afforded *O*-tosylated compound as white solid. This tosyl AZT compound was dissolved in 80 mL of DMF, to which $NaN_3$ was added (1.4 g, 22.2 mmol) and heated at 60 °C for 12 h. Then the reaction was quenched by the addition of water (200 mL) and extracted in ethyl acetate (2 x 75 mL). The combined organic layer was dried over $Na_2SO_4$, concentrated and purified by column chromatography to get pure product 2 (0.96 g, 88% yield) as a colorless viscous liquid. $^1H$ NMR (500 MHz, $CDCl_3$): δ 8.73 (brs, 1H), 7.30 – 7.27 (m, 1H), 6.14 (t, *J* = 6.0 Hz, 1H), 4.25 (dt, *J* = 7.7, 5.5 Hz, 1H), 3.94 (dt, *J* = 5.5, 3.4 Hz, 1H), 3.77 (dd, *J* = 13.4, 3.3 Hz, 1H), 3.57 (dd, *J* = 13.4, 3.5 Hz, 1H), 2.49 – 2.36 (m, 2H), 1.94 (d, *J* = 1.1 Hz, 3H). $^{13}C$ NMR (100 MHz, $CDCl_3$): δ 163.9, 150.5, 135.6, 111.8, 85.2, 82.2, 60.5, 51.9, 37.4, 12.7. MS (MALDI-TOF): calcd for $C_{10}H_{12}N_8O_3$: 292.1, found 293.1 $(M+H)^+$.

**1-((2R,4S,5R)-4-azido-5-(azidomethyl)tetrahydrofuran-2-yl)-5-methyl-4-(1H-1,2,4-triazol-1-yl)pyrimidin-2(1H)-one (3):** To ice-cold stirring solution of $POCl_3$ (5.1 mL, 54.7 mmol), TEA (57 mL, 410.9 mmol) and 1,2,4-triazole (17.0 g, 246.5 mmol) in anhydrous acetonitrile (150 mL) under $N_2$ atmosphere was added compound 2 (8 g, 27.3 mmol) in 80 mL of anhydrous acetonitrile in dropwise. Then the reaction mixture was stirred at room temperature for 20 h. It was then extracted with ethyl acetate (200 mL x 2). The combined organic phase was washed with saturated $NaHCO_3$ solution (200 mL), brine (150 mL) and dried over $Na_2SO_4$, concentrated and purified by column chromatography to afford pure product 3 (9.2 g, 98% yield) as a white solid. $^1H$ NMR (500 MHz, $CDCl_3$): δ 9.27 (s, 1H), 8.15 (s, 1H), 8.11 (s, 1H), 6.15 (dd, *J* = 6.7, 4.7 Hz, 1H), 4.18 (q, *J* = 6.8 Hz, 1H), 4.07 – 4.02 (m, 1H), 3.90 (dd, *J* = 13.5, 3.1 Hz, 1H), 3.67 (dd, *J* = 13.5, 3.2 Hz, 1H), 2.79 – 2.70 (m, 1H),

2.49 – 2.43 (m, 4H). $^{13}$C NMR (100 MHz, $CDCl_3$): δ 158.5, 153.7, 153.7, 146.3, 145.2, 106.0, 87.3, 82.8, 59.4, 51.4, 38.7, 17.5. MS (MALDI-TOF): calcd for $C_{12}H_{13}N_{11}O_2$: 343.1, found 344.1 $(M+H)^+$.

**Di-tert-butyl(((1-((2R,4S,5R)-4-azido-5-(azidomethyl)tetrahydrofuran-2-yl)-5-methyl-2-oxo-1,2-dihydropyrimidin-4-yl)azanediyl)bis(propane-3,1-diyl))dicarbamate (5)**

To a stirred solution of $N^1$,$N^9$-bis-Boc-norspermidine 4, (2.3 g, 7.0 mmol) in acetonitrile (30 mL) was added $K_2CO_3$ (2.4 g, 17.4 mmol) followed by compound 3 ( 2 g, 5.8 mmol) at room temperature. Then the reaction mixture was stirred for 24 h at 40 °C. After completion of reaction, solvent was removed through vacuum. The $H_2O$ (20 mL) was added to the crude product and extracted with 2% methanol in ethylacetate (20 mL x 3). The combine organic layer was washed with brine (30 mL), dried over $Na_2SO_4$ and concentrated, and purified by flash chromatography to obtain pure compound 5 (3.0 g, 87% yield) as white sticky solid. $^1$H NMR (500 MHz, $CDCl_3$) δ 7.35 (s, 1H), 6.11 (t, $J$ = 6.0 Hz, 1H), 5.05 (brs, 2H), 4.20 (q, $J$ = 6.6 Hz, 1H), 3.95 – 3.89 (m, 1H), 3.79 – 3.74 (m, 1H), 3.65 – 3.53 (m, 5H), 3.17 – 3.05 (m, 4H), 2.57 – 2.47 (m, 1H), 2.47 – 2.36 (m, 1H), 2.16 (s, 3H), 1.87 – 1.74 (m, 4H), 1.42 (s, 18H). $^{13}$C NMR (100 MHz, $CDCl_3$)δ 164.3, 156.3, 154.1, 141.3, 102.8, 86.2, 82.2, 79.4, 60.3, 51.8, 47.5, 38.1, 38.1, 29.2, 28.5, 19.0. MS (MALDI-TOF): calcd for $C_{26}H_{43}N_{11}O_6$: 605.3; found 606.2 $(M+H)^+$.

**di-tert-butyl(((1-((2R,4S,5R)-4-(2-((3S,5S,7S)-adamantan-1-yl)acetamido)-5-((2-((3R,5R,7R)-adamantan-1-yl)acetamido)methyl)tetrahydrofuran-2-yl)-5-methyl-2-oxo-1,2-dihydropyrimidin-4-yl)azanediyl)bis(propane-3,1-diyl))dicarbamate (6)**

Compound 5 (984 mg, 1.62 mmol) was dissolved in methanol (20 mL), to which 54 mg of 5% (Pd/C) was added and stirred for 6 h under $H_2$ atm. After completion of reaction, filtered through celite pad and concentrate residue to get crude diamine compound which was proceeded to next step directly. To a stirred solution of 1-adamantaneacetic acid (663 mg, 3.41 mmol) in dimethylformamide (15 mL) was added subsequently HOBt (370 mg, 2.7 mmol) and EDC HCl (935 mg, 4.88 mmol) under $N_2$ atm. After stirring 10 min, diamine (900 mg, 1.62 mmol) in dimethylformamide (5 mL) and DIPEA (1.5 mL, 8.13 mmol) was added and stirred for 10 h at room temperature. After dilution with $CH_2Cl_2$ (50 mL), the mixture was washed with 2% citric acid (15 mL), $H_2O$ (15 mL), 5% aq. $NaHCO_3$ soln. (15 mL), and brine. The combined organic layeres were

dried ($Na_2SO_4$) and concentrated, and the crude residue was purified by column chromatography using MeOH:$CH_2Cl_2$ (2:98) to afford the pure product 13a as a white solid (980 mg, 86% yield). $^1$H NMR (500 MHz, $CDCl_3$) δ 7.46 (brs, 1H), 7.34 (s, 1H), 7.18 (brs, 1H), 6.25 – 6.12 (m, 1H), 5.07 (brs, 2H), 4.19 – 4.11 (m, 1H), 4.00 – 3.93 (m, 1H), 3.64 – 3.54 (m, 5H), 3.46 – 3.39 (m, 1H), 3.15 – 3.07 (m, 4H), 2.20 – 2.12 (m, 4H), 2.01 – 1.96 (m, 3H), 1.95 – 1.91 (m, 6H), 1.82 – 1.75 (m, 4H), 1.69 – 1.54 (m, 26H), 1.42 (s, 18H). $^{13}$C NMR (100 MHz, $CDCl_3$) δ 172.6, 171.8, 164.4, 156.3, 154.8, 141.0, 103.1, 85.7, 84.4, 79.4, 51.8, 51.7, 50.8, 47.5, 42.7, 42.0, 38.1, 37.3, 36.9, 36.8, 33.0, 32.9, 29.2, 28.8, 28.7, 28.5, 19.0. MS (MALDI-TOF): calcd for $C_{50}H_{79}N_7O_8$: 905.6; found 928.5 $(M+Na)^+$.

**2-((3R,5R,7R)-adamantan-1-yl)-N-(((2R,3S,5R)-3-(2-((3S,5S,7S)-adamantan-1-yl)acetamido)-5-(4-(bis(3-aminopropyl)amino)-5-methyl-2-oxopyrimidin-1(2H)-yl)tetrahydrofuran-2-yl)methyl)acetamide (7)**

Boc- protected intermediate 6 (910 mg, 1.0 mmol) was dissolved in 1.25M HCl in MeOH (10 mL) and stirred at 50 °C for 1 h. The reaction mixture was concentrated under reduced pressure. The crude was washed with acetonitrile and diethylether (1:1), followed by 10% ethyl acetate in hexane to afford the product 7 as white solid (645 mg, 83%). %). $^1$H NMR (800 MHz, DMSO-$d_6$) δ 8.21 (d, $J$ = 7.2 Hz, 1H), 7.92 – 7.88 (m, 5H), 7.64 (s, 1H), 6.12 (t, $J$ = 6.6 Hz, 1H), 4.19 – 4.16 (m, 1H), 3.79 – 3.77 (m, 1H), 3.55 – 3.49 (m, 4H), 3.40 – 3.36 (m, 1H), 3.30 – 3.27 (m, 1H), 2.83 – 2.78 (m, 4H), 2.19 (s, 3H), 2.16 – 2.12 (m, 2H), 1.91 – 1.89 (m, 4H), 1.86 – 1.84 (m, 6H), 1.66 – 1.62 (m, 6H), 1.58 – 1.52 (m, 22H). $^{13}$C NMR (200 MHz, DMSO-$d_6$): δ 170.1, 170.0, 158.2, 153.5, 142.1, 116.6, 102.0, 84.8, 83.0, 50.5, 50.0, 49.7, 45.7, 42.1, 42.0, 39.8, 36.4, 32.3, 32.2, 28.0, 26.2, 17.5, 12.7. MALDI-TOF): calcd for $C_{40}H_{63}N_7O_4$: 705.5, found 706.5 $(M+H)^+$.

**2-((3R,5R,7R)-adamantan-1-yl)-N-(((2R,3S,5R)-3-(2-((3S,5S,7S)-adamantan-1-yl)acetamido)-5-(4-(bis(3-guanidinopropyl)amino)-5-methyl-2-oxopyrimidin-1(2H)-yl)tetrahydrofuran-2-yl)methyl)acetamide (ADG-2e)**

1*H*-Pyrazole-1-carboxamidine hydrochloride (75 mg, 0.51 mmol) was added to a solution of 7 (120 mg, 0.17 mmol) and DIPEA (0.23 mL, 1.02 mmol) in dimethylformamide (5 mL), and stirred for

overnight. The reaction mixture was concentrated under vacuum, dissolved in 1.25M HCl in MeOH (8 mL), and stirred for 1 h at 0 °C. Solvent was evaporated and treated with ethylacetate and acetonitrile mixture (4:6)
of 10 ml. The resultant solid was further washed with 10 ml diethylether to afford the final product as white solid. (110 mg, 82 %). $^{1}$H NMR (800 MHz, DMSO-$d_6$) δ 8.38 (brs, 2H), 8.21 (d, *J* = 7.1 Hz, 1H), 7.89 (t, *J* = 5.8 Hz, 1H), 7.68 (s, 1H), 7.35 (brs, 6H), 6.14 (t, *J* = 6.6 Hz, 1H), 4.18 – 4.14 (m, 1H), 3.81 – 3.78 (m, 1H), 3.54 – 3.48 (m, 4H), 3.44 – 3.40 (m, 1H), 3.30 – 3.25 (m, 1H), 3.14 (q, *J* = 5.8 Hz, 4H), 2.20 (s, 3H), 2.19 – 2.14 (m, 2H), 1.91 – 1.81 (m, 13H), 1.66 – 1.50 (m, 25H). $^{13}$C NMR (200 MHz, DMSO-$d_6$): 170.1, 170.0, 163.0, 158.4, 156.9, 154.4, 141.9, 116.4, 102.9, 84.8, 83.1, 50.6, 50.0, 49.7, 47.0, 42.1, 42.0, 41.2, 39.9, 39.8, 39.7, 39.6, 38.8, 36.7, 36.4, 32.3, 32.2, 28.0, 17.6. MS (MALDI-TOF): calcd for $C_{42}H_{67}N_{11}O_4$: 789.5, found 790.5 $(M+ H)^+$.

**Physical and chemical properties of ADG-2e**

Molecular weight: 789, cLogp:1.45, TPSA:227, solubility:-5.7 (OSIRIS Property Explorer (https://www.organic-chemistry.org/prog/peo/)

**Reference**


1. Chirumarry, S.; Soung, N.-K.; Han, J.; Kim, E.Y.; Ryu, E.K.; Lee, Y.-H.; Shin, S.Y.; Gunasekaran, P.; Bang, J.K. Antibacterial AZT derivative regulates metastasis of breast cancer cells. *Eur J Med Chem* **2020**, *193*, 112233, doi:https://doi.org/10.1016/j.ejmech.2020.112233.
